# Epidemiology of Plasmid Lineages Mediating the Spread of Extended-Spectrum Beta-Lactamases among Clinical Escherichia coli

**DOI:** 10.1128/msystems.00519-22

**Published:** 2022-08-22

**Authors:** Bejan Mahmud, Meghan A. Wallace, Kimberly A. Reske, Kelly Alvarado, Carol E. Muenks, David A. Rasmussen, Carey-Ann D. Burnham, Cristina Lanzas, Erik R. Dubberke, Gautam Dantas

**Affiliations:** a The Edison Family Center for Genome Sciences & Systems Biology, Washington University School of Medicine, St. Louis, Missouri, USA; b Department of Pathology and Immunology, Washington University School of Medicine, St. Louis, Missouri, USA; c Department of Medicine, Division of Infectious Diseases, Washington University School of Medicine, St. Louis, Missouri, USA; d Department of Entomology and Plant Pathology, North Carolina State University, Raleigh, North Carolina, USA; e Bioinformatics Research Center, North Carolina State University, Raleigh, North Carolina, USA; f Department of Molecular Microbiology, Washington University School of Medicine, St. Louis, Missouri, USA; g Department of Pediatrics, Washington University School of Medicine, St. Louis, Missouri, USA; h Department of Population Health and Pathobiology, College of Veterinary Medicine, North Carolina State University, Raleigh, North Carolina, USA; i Department of Biomedical Engineering, Washington University in St. Louis, St. Louis, Missouri, USA; Marquette University

**Keywords:** *Escherichia coli*, beta-lactamases, plasmid-mediated resistance

## Abstract

The prevalence of extended-spectrum beta-lactamases (ESBLs) among clinical isolates of Escherichia coli has been increasing, with this spread driven by ESBL-encoding plasmids. However, the epidemiology of ESBL-disseminating plasmids remains understudied, obscuring the roles of individual plasmid lineages in ESBL spread. To address this, we performed an in-depth genomic investigation of 149 clinical ESBL-like E. coli isolates from a tertiary care hospital. We obtained high-quality assemblies for 446 plasmids, revealing an extensive map of plasmid sharing that crosses time, space, and bacterial sequence type boundaries. Through a sequence-based network, we identified specific plasmid lineages that are responsible for the dissemination of major ESBLs. Notably, we demonstrate that IncF plasmids separate into 2 distinct lineages that are enriched for different ESBLs and occupy distinct host ranges. Our work provides a detailed picture of plasmid-mediated spread of ESBLs, demonstrating the extensive sequence diversity within identified lineages, while highlighting the genetic elements that underlie the persistence of these plasmids within the clinical E. coli population.

**IMPORTANCE** The increasing incidence of nosocomial infections with extended-spectrum beta-lactamase (ESBL)-producing Escherichia coli represents a significant threat to public health, given the limited treatment options available for such infections. The rapid ESBL spread is suggested to be driven by localization of the resistance genes on conjugative plasmids. Here, we identify the contributions of different plasmid lineages in the nosocomial spread of ESBLs. We provide further support for plasmid-mediated spread of ESBLs but demonstrate that some ESBL genes rely on dissemination through plasmids more than the others. We identify key plasmid lineages that are enriched in major ESBL genes and highlight the encoded genetic elements that facilitate the transmission and stable maintenance of these plasmid groups within the clinical E. coli population. Overall, our work provides valuable insight into the dissemination of ESBLs through plasmids, furthering our understating of factors underlying the increased prevalence of these genes in nosocomial settings.

## INTRODUCTION

Since their first report in the 1980s, extended-spectrum beta-lactamase (ESBL)-producing *Enterobacterales* have spread globally; it is now estimated that approximately 1.5 billion people are colonized with such organisms (1). Infections with ESBL-producing *Enterobacterales* are associated with a higher likelihood of treatment failure, higher mortality, and added financial burdens on the patients and the healthcare system (2). While historically the TEM and SHV ESBL families were the predominant genes found in infectious isolates, the CTX-M group of ESBLs has emerged more recently as the most prevalent and has driven the global ESBL dissemination (3, 4). Escherichia coli is the primary carrier of the CTX-M family of ESBLs (3), and the rates of community carriage of ESBL-producing E. coli are on the rise, particularly in low- and middle-income countries (5). The spread of ESBL E. coli in nosocomial settings is even more pervasive: the rates of ESBL production among clinical E. coli isolates have steadily increased globally over the years, with the reported ESBL rates among hospital-associated infections in the US being as high as 27.7% (4, 6, 7).

The widespread dissemination of the CTX-M gene family in health care settings is attributed to the association of these genes with mobile genetic elements (MGEs) (5) in this environment with high antibiotic selective pressure. Of particular interest are the conjugative IncF family of plasmids, which have been reported as one of the primary culprits for the spread of ESBLs (8–11). Consequently, various plasmid curing mechanisms, such as conjugation inhibitors, have been proposed to be used clinically to ease the ESBL resistance burden (12, 13). However, the potential benefits of such proposals remain partially obscured by the understudied epidemiology of ESBL-encoding plasmids. Specifically, while numerous studies have successfully described plasmids carrying ESBLs, such efforts have typically focused on epidemiologically unrelated plasmids (14–19), had a relatively small plasmid set (14, 20–24), or were methodologically limited in their resolution (15–18, 21, 25–28). As such, questions remain regarding the dynamics of plasmids mediating the spread of ESBLs within defined health care systems. Specifically, the extent of plasmid sharing within a clinical E. coli population, the roles of different plasmid lineages in the local spread of individual ESBLs, and the long-term genetic stabilities of such lineages remain underexplored.

To address the above knowledge gaps, we conducted an in-depth, high-resolution, genomic analysis of 149 clinical ESBL E. coli isolates collected over a 3-year period from a single tertiary-care hospital. We identified an extensive network of plasmid sharing that crosses time, space, and phylogenetic barriers. IncF plasmids were found to be significantly enriched in ESBL genes, constituting 2 distinct plasmid lineages that are associated with different ESBL genes and occupy varying host ranges. However, plasmid-mediated resistance spread was not uniform, as certain ESBLs were predominantly chromosomally encoded and may thus rely more on bacterial host clonal expansion for their dissemination.

## RESULTS

### Sample description.

**FIG 1:**
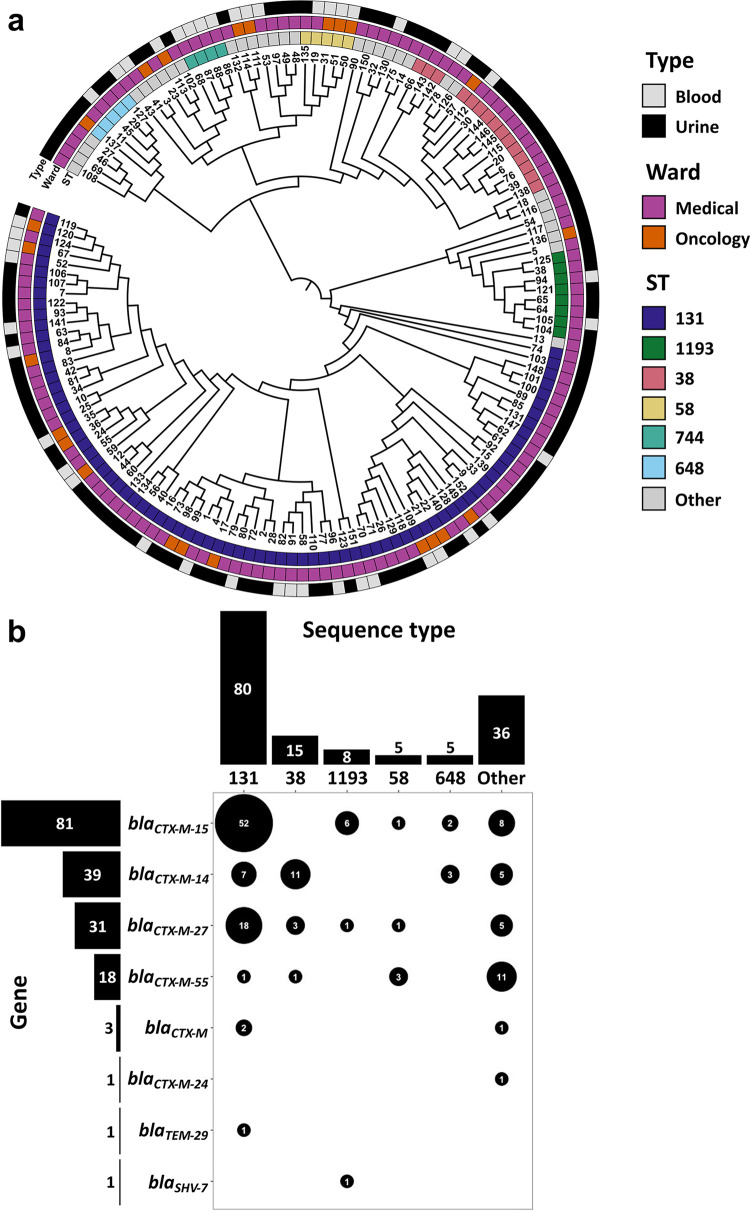
Abundance of E. coli sequence types (STs) and extended-spectrum beta-lactamases (ESBLs). (a) Maximum likelihood phylogenetic tree from the alignment of 2,888 core genes of the 149 ESBL E. coli isolates. The outer rings are colored to indicate the major STs, collection wards, and the types of originating clinical specimens. Sequence typing of the isolates was done using the Achtman scheme. (b) Abundances of ESBL genes and major STs are shown as bar plots. The identified ST-ESBL pairs are shown in circles, and the sizes of the circles are proportional to the number of instances of the given pair identified in our isolate set; the ST-ESBL pair counts are indicated in the circles. The isolates containing multiple copies of the same gene were counted only once toward the corresponding ST-ESBL pair.

A total of 367 blood or urine specimens that grew ESBL E. coli were collected during the study period from patients admitted to the medical and/or oncology wards and intensive care units of the Barnes-Jewish Hospital between June 2016 and December 2019. After excluding duplicate specimens collected within 14 days of an initial specimen, 313 specimens were eligible for inclusion (see Materials and Methods). Of these, remnant specimens were available for 149 E. coli isolates (47.6% of the total eligible specimens), originating from a cohort of 129 patients; most of the patients contributed only a single isolate (see Materials and Methods) (Table S1). All isolates displayed phenotypic susceptibility patterns characteristic of ESBL-producing strains, namely, resistant to at least one third generation cephalosporin and susceptible to cephamycins (see Materials and Methods). Phylogenetic clustering of isolates by specimen type (urine versus blood) and collection ward (medical versus oncology) was not observed (Fig. 1a). Multi-locus sequence typing using the Achtman scheme (29) identified 27 distinct sequence types (STs) (Fig. S1). Consistent with the previous reports of its global prevalence (30, 31), ST131 was most frequently recovered from our patient cohort (Fig. 1b and Fig. S1).

10.1128/msystems.00519-22.1 TABLE S1 Isolates and patients involved in the study. Download Table S1, DOCX file, 0.01 MB. Copyright © 2022 Mahmud et al. 2022 Mahmud et al. https://creativecommons.org/licenses/by/4.0/ This content is distributed under the terms of the Creative Commons Attribution 4.0 International license.

10.1128/msystems.00519-22.3 FIG S1 Identified ST-ESBL pairs. Identified STs and ESBLs, and their corresponding counts are shown as bar plots. Sequence typing of the isolates was done using the Achtman scheme; untyped isolates are shown as U. Identified ST-ESBL pairs are shown as circles, with the sizes being proportional to the number of corresponding pairs identified. The ESBL-ST counts are indicated in the circles. Download FIG S1, TIF file, 0.4 MB. Copyright © 2022 Mahmud et al. 2022 Mahmud et al. https://creativecommons.org/licenses/by/4.0/ This content is distributed under the terms of the Creative Commons Attribution 4.0 International license.

### Variability in genetic origins of ESBLs.

Overall, we identified 8 distinct ESBL genotypes in our isolates (Fig. 1b). Among identified ESBLs, *bla_CTX-M-15_* was the most common (Fig. 1b and Fig. S1). Similarly, the *bla_CTX-M-15_*-ST131 pairing was most common in our data set; however, significant enrichment of the gene in ST131 hosts was not observed (*P* = 0.36, Chi-Square goodness of fit, Bonferroni correction for multiple comparisons). Instances of isolates carrying 2 distinct ESBL genes were uncommon but present, with 2 isolates encoding both *bla_CTX-M-15_* and *bla_CTX-M-14_*, another isolate encoding for *bla_CTX-M-27_* and *bla_CTX-M-55_*, and the pair of *bla_CTX-M-15_* and *bla_CTX-M_* found in yet another isolate (Data Set S1, Sheet A). Despite displaying an ESBL-like phenotype (see Materials and Methods), no known ESBL genes were identified in 8 of the isolates (Data Set S1, Sheet A).

The genetic origin (chromosomal versus plasmidic) of resistance genes is a major determinant of transmission routes (vertical versus horizontal) and has been noted to affect antibiotic susceptibility in the bacterial host (32). Accordingly, we investigated the distribution of ESBLs between chromosomal and plasmidic contigs in our cohort (Table S2). The genetic origins of the major ESBL genotypes varied considerably. *bla_CTX-M-14_* was spread evenly between the 2 genetic elements. *bla_CTX-M-15_* was predominantly encoded chromosomally, whereas *bla_CTX-M-27_* and *bla_CTX-M-55_* were predominantly found in plasmids (Table S2).

10.1128/msystems.00519-22.2 TABLE S2 Genetic origin (chromosomal vs. plasmidic) of the identified extended-spectrum beta-lactamases. Download Table S2, DOCX file, 0.01 MB. Copyright © 2022 Mahmud et al. 2022 Mahmud et al. https://creativecommons.org/licenses/by/4.0/ This content is distributed under the terms of the Creative Commons Attribution 4.0 International license.

### Amplification of *bla_CTX-M-14_* within a persistent E. coli lineage.

**FIG 2:**
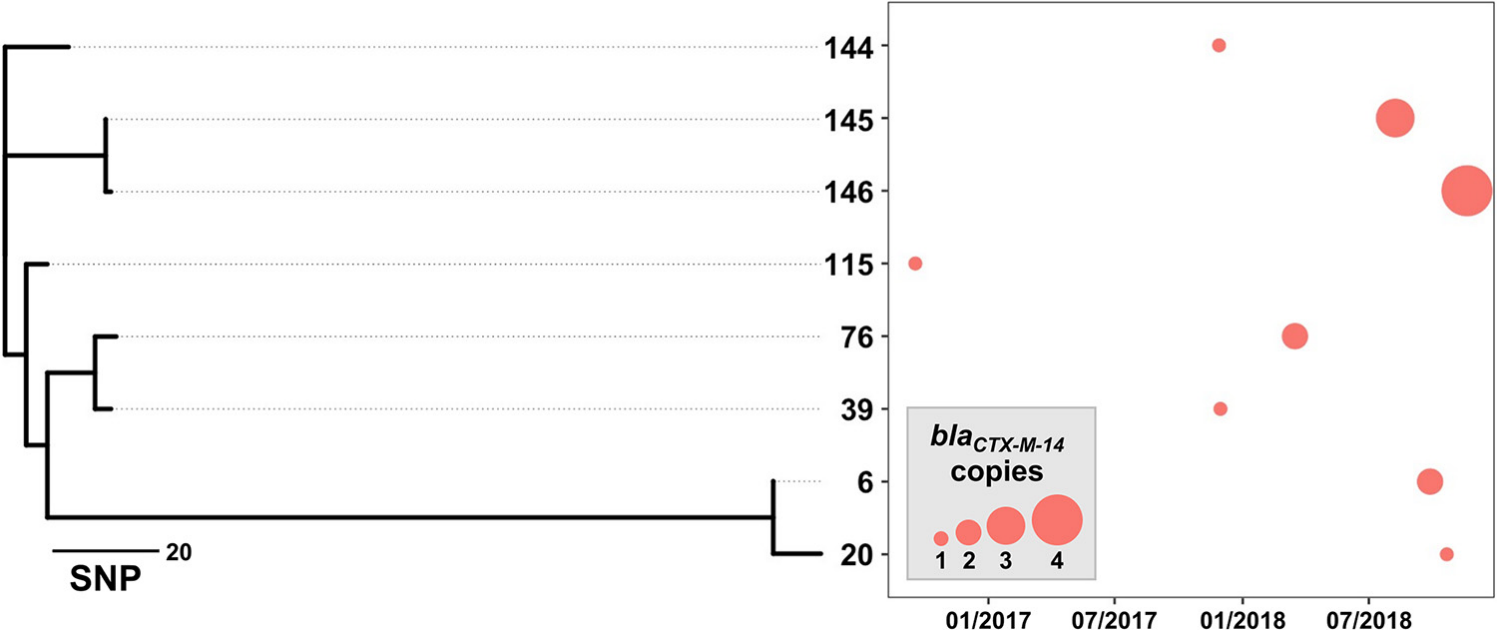
Persistence of a *bla_CTX-M-14_*-encoding E. coli lineage. The left panel displays a phylogenetic tree of the 8 isolates identified to be highly related. Relatedness is determined through the number of single-nucleotide polymorphisms (SNPs) across 4,925,981 chromosomal core sites, defined as sites present in all isolates. The branch lengths indicate the SNP distances among isolates. All pairs of isolates are within 174 SNPs of each other. The right panel shows the collection dates (*x* axis) for the 8 isolates, denoted by red circles. The size of the circles corresponds to the number of *bla_CTX-M-14_* gene copies found within the given isolate. Isolates 144, 145, and 146 were isolated from the same patient; other isolates originated from distinct patients.

Several isolates in our set carried multiple copies of a given ESBL gene. The presence and biological significance of multiple ESBL gene copies in E. coli isolates have been noted before (33–35). Specifically, isolates with multiple copies of *bla_CTX-M-15_*, *bla_CTX-M-14_*, *bla_CTX-M-27_*, or *bla_CTX-M-55_* were identified in our set (Data Set S1, Sheet A). Most notably, isolate 146 was found to carry 4 copies of *bla_CTX-M-14_*, 3 encoded in distinct and distant chromosomal loci and 1 copy carried in a plasmid (Fig. 2 and Fig. S2b). Interestingly, isolate 146 was the latest of the 3 isolates (144-146) cultured across a year-long period from urine samples of the same patient. These isolates likely constitute a single lineage, and all carry a 75-Kb plasmid encoding *bla_CTX-M-14_* (Fig. 2 and Fig. S2a). However, the isolates differ in the number of chromosomal copies of *bla_CTX-M-14_*, with 2 copies in 145 and none in 144, suggesting that this lineage has experienced an enrichment in the *bla_CTX-M-14_* copy number over the course of the disease. Furthermore, isolates from the same lineage were also identified in urine samples collected over a 2-year period from other patients (Fig. 2), and all similarly carry the aforementioned *bla_CTX-M-14_*-encoding 75-Kb plasmid (Fig. S2a). Chromosomal copies of *bla_CTX-M-14_* were also identified in 2 other isolates (Fig. 2), suggesting that resistance gene amplification is not limited to the patient harboring isolates 144–146 and is a more general feature of this lineage. These data demonstrate the clinical persistence of an infectious E. coli lineage, and dissemination and amplification of an ESBL through association with this lineage.

10.1128/msystems.00519-22.4 FIG S2 Carriage of *bla_CTX-M-14_* within conserved genetic contexts. In both panels (a) and (b) BLASTn similarity scores of 100% (rounded to the nearest integer) are colored in accordance with the matching orientation (i.e., forward or reverse). Protein annotations are shown in arrows. *bla_CTX-M-14_* and relevant nearby open reading frames are colored. (a) 75-Kb circular contigs identified in assemblies of the isolates identified in Fig. 2. (b) Genetic context of the *blaCTX-M-14* copies found in isolates 144-146. The chromosomal (C) or plasmidic (P) origin of each illustrated gene copy is indicated. Download FIG S2, TIF file, 1.1 MB. Copyright © 2022 Mahmud et al. 2022 Mahmud et al. https://creativecommons.org/licenses/by/4.0/ This content is distributed under the terms of the Creative Commons Attribution 4.0 International license.

### ESBLs are commonly encoded within conserved transposable elements.

**FIG 3:**
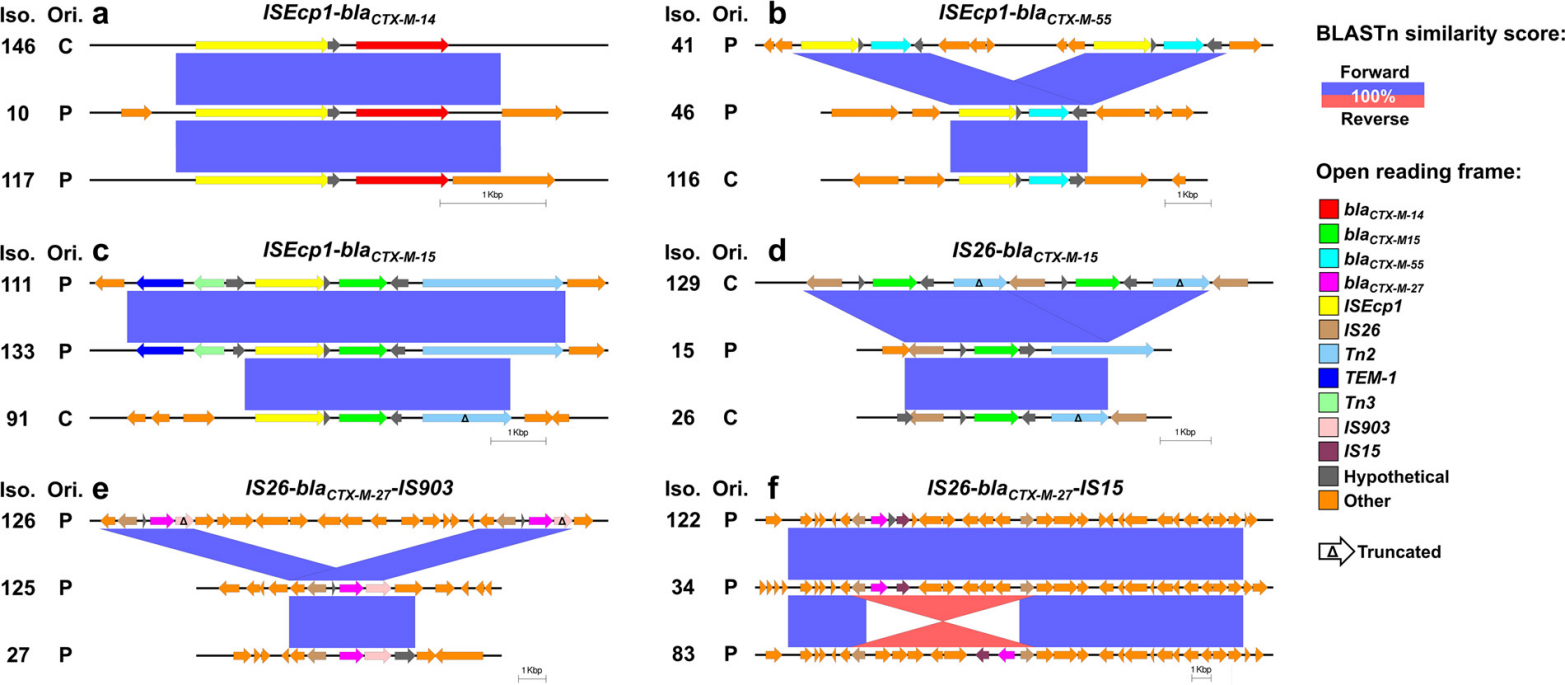
Transposases associated with major extended-spectrum beta-lactamases (ESBLs). Conserved genetic cassettes encoding *bla_CTX-M-14_* (a), *bla_CTX-M-55_* (b), *bla_CTX-M-15_* (c,d), and *bla_CTX-M-27_* (e,f). BLASTn similarity scores of 100% (rounded to the nearest integer) colored in accordance with the matching orientation (i.e., forward or reverse). Protein annotations are shown in arrows. ESBLs and relevant nearby open reading frames are colored. Truncated open reading frames are indicated with Δ. The chromosomal (C) or plasmidic (P) origins of the genetic elements are indicated.

ESBL genes were often encoded near transposases within conserved cassettes. Analysis of the *bla_CTX-M-14_* genomic context within chromosomal and plasmidic copies of the gene found in isolates 144, 145, and 146 identified the gene within a conserved cassette encoding the *ISEcp1* transposase upstream of the gene in all instances (Fig. S2b). Such genetic organization was observed in other, distantly related isolates carrying *bla_CTX-M-14_* (Fig. 3a), with the gene located downstream of *ISEcp1* in 92.3% (36/39) of the instances in our data set. Similarly, *bla_CTX-M-55_* was most commonly (56.4%, 11/18) observed to be encoded within a conserved cassette downstream of the *ISEcp1* (Fig. 3b); *bla_CTX-M-55_* was often (33.3%, 6/18) found downstream of *IS15* as well. *ISEcp1* was also associated with *bla_CTX-M-15_*, which was typically (65.4%, 53/81) found to be located within a conserved unit downstream of the transposase (Fig. 3c). This *ISEcp1*-*bla_CTX-M-15_* configuration is seldom found within a larger conserved window that also encodes for the *TEM-1* broad-spectrum beta-lactamase and the *Tn3* resolvase (36). Such prevalent association of *ISEcp1* with 3 of the 4 most common ESBL genotypes suggests that the transposase is predominantly responsible for the dissemination of ESBLs within our clinical population.

Another notable transposase associated with *bla_CTX-M-15_* is *IS26*, which was commonly (29.6%, 24/81) found upstream of the resistance gene (35) (Fig. 3d). *IS26* may also drive the mobilization of *bla_CTX-M-27_* within our clinical isolates, as the transposase was identified upstream of *bla_CTX-M-27_* in 83.9% (26/31) of instances, commonly encoding *IS903* (35.5%, 11/31) (Fig. 3e) or *IS15* (38.7%, 12/31) (Fig. 3f) downstream of the resistance gene. The latter conformation, *IS26*-*bla_CTX-M-27_*-*IS15*, was located within a conserved ~20-Kb window (37). An *IS26*-flanked sub-region within this window was found inverted in 1 of our plasmids, suggesting its capacity for mobilization independent of the 20-Kb segment (Fig. 3f). All major ESBL genotypes found in our set are commonly encoded within conserved windows proximal to transposases (primarily *ISEcp1* and *IS26*), which have likely contributed to within-isolate gene multiplication events and facilitated the within-population spread of the resistance genes (i.e., through transposition to a plasmid).

### Sharing of plasmids across space, time, and genetic barriers.

**FIG 4:**
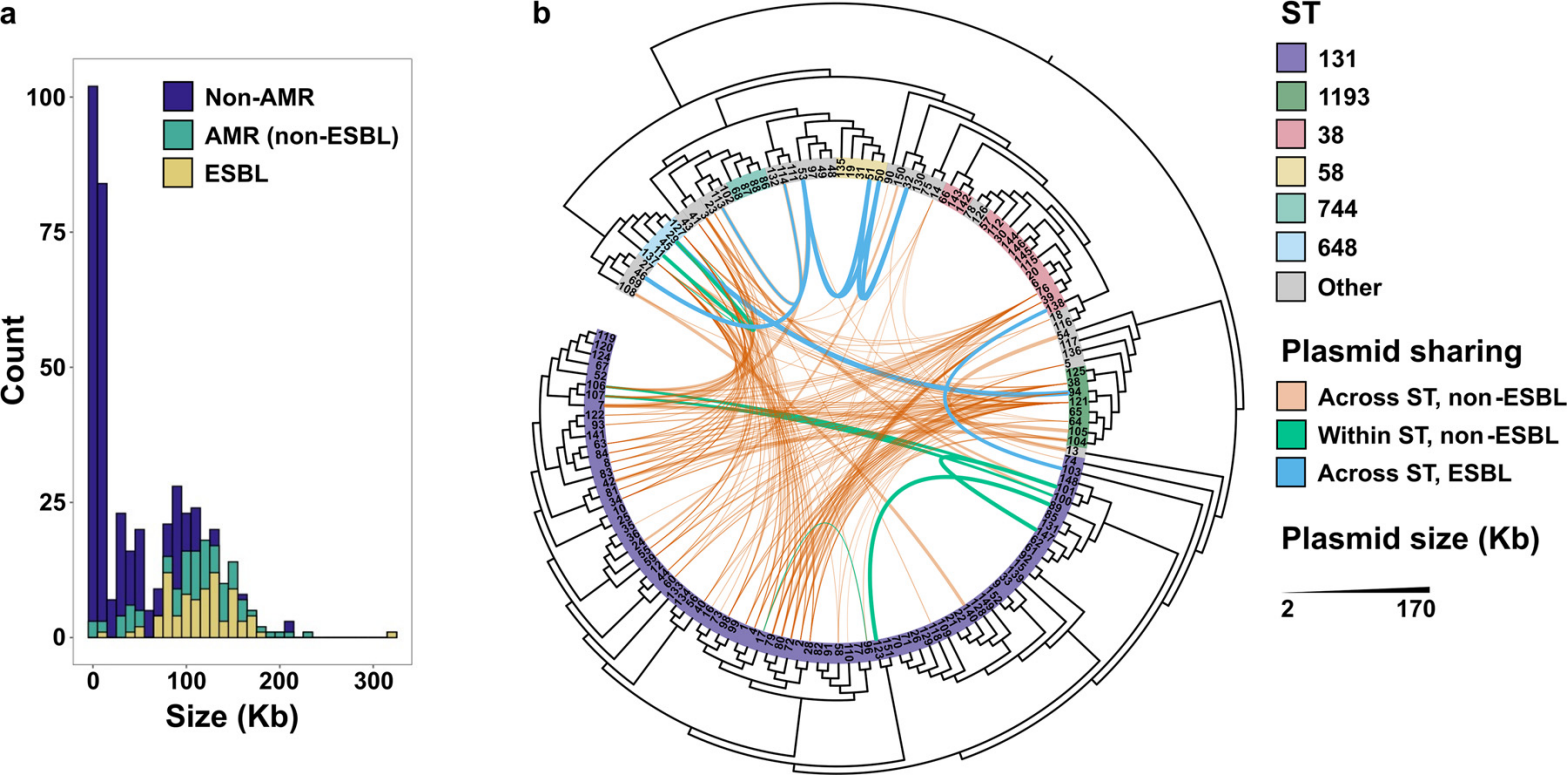
Plasmids carried by extended-spectrum beta-lactamase (ESBL)-encoding E. coli isolates. (a) Histogram depicting the distribution of assembled plasmidic contigs by size. The fractions of plasmids encoding ESBLs, non-ESBL antimicrobial (AMR) genes, and lacking AMR genes in each bin are colored. Bin size = 10 Kb. (b) Maximum likelihood phylogenetic tree of the 149 ESBL E. coli isolates. Isolates are colored by major sequence types (STs). The inner lines connect isolate pairs with >550 single-nucleotide polymorphisms (2,888 core genes; Fig. S3), in which near-identical pair of plasmids (i.e., shared plasmids) were identified. Two plasmids were defined to be near-identical if they i) had ≥95% coverage and ≥99% identity by BLASTn, and ii) did not differ in size by more than 10% of the larger of the two plasmids. The connecting lines are colored to indicate sharing of non-ESBL plasmids within and across ST, and sharing of ESBL-encoding plasmids within ST. Thickness of the lines corresponds to the average of the sizes of the two plasmids.

Plasmids are the predominant units of horizontal transmission of ESBLs and are likely responsible for their rapid global spread (38). We conducted an in-depth analysis of the total plasmid content of our isolate set to identify genetic patterns associated with the spread of ESBLs. Contigs were labeled as plasmidic if they were: i) under 1M-bp in length and ii) circular. We identified 446 plasmidic contigs, with the mode of 2 plasmids/isolate and a range of 1–10 plasmids/isolate. The identified plasmid sizes ranged from 1,547 bp to 320,261 bp and displayed a multimodal distribution, with a large number of plasmids under 20 Kb in size and mostly devoid of antimicrobial resistance genes (Fig. 4a).

10.1128/msystems.00519-22.5 FIG S3 Distribution of pairwise core single-nucleotide polymorphisms (SNPs) among the 149 E. coli isolates. SNP counts were based on the alignment of 2,888 core genes (present in ≥99% of the isolates). The area outlined in dashed, red line is shown in the inner plot. Dashed black line (550 SNPs) indicates the lower threshold for defining distantly related isolates. Bin sizes are 2,286 and 25 for the outer and inner histograms, respectively. Download FIG S3, JPG file, 0.3 MB. Copyright © 2022 Mahmud et al. 2022 Mahmud et al. https://creativecommons.org/licenses/by/4.0/ This content is distributed under the terms of the Creative Commons Attribution 4.0 International license.

We identified numerous instances of plasmid sharing between distantly related isolates (>550 core single-nucleotide polymorphisms [SNPs]) (Fig. S3). Plasmids were compared via BLASTn in all pairwise combinations, and the hits with ≥95% coverage, ≥99% identity, and not differing in size by more than 10% of the larger of the 2 plasmids in the pair were considered as potential instances of plasmid sharing. Furthermore, to account for acquisition/loss of transposable fragments by plasmids (Fig. S4a) and to minimize the rate of false matches, we only considered plasmid pairs where the corresponding sizes did not differ by more than 10% of the larger value. We identified an extensive network of plasmid sharing within our sample set (Fig. 4b). Sharing of plasmids across STs is common, with 31 unique ST pairs identified in this network. Near-identical plasmids were found in isolates collected as long as 3 years apart from distinct patients (mean of 383 days between collections); moreover, a quarter of such plasmids were present in isolates from both medical and oncology wards. We also observed sharing of ESBL-encoding plasmids by phylogenetically distant isolates (Fig. 4b). Notably, near-identical *bla_CTX-M-55_*-encoding plasmids were present in urine isolates (103 and 138) collected a year apart from distinct patients (Fig. S4a). Numerous instances of phylogenetically distant isolates carrying multiple near-identical plasmids were also identified. Most notably, blood isolates 113 and 114, collected 2 months apart from the same patient, both carried a *bla_CTX-M-55_*-encoding 48-Kb and a 103-Kb plasmids (Fig. S4b and c). Our network analysis of shared plasmids demonstrates the persistence of plasmids across space and time within a diverse set of clinical hosts, and commonplace plasmid sharing unrestricted by ST boundaries.

10.1128/msystems.00519-22.6 FIG S4 Relatedness of the identified plasmids. (a–c) Near-identical plasmids found in distantly related isolates. Instances of plasmid sharing between isolates 103 and 138 (a) and isolates 113 and 114 (b,c). BLASTn similarity scores of at least 91% (rounded to the nearest integer) are colored in accordance with the matching orientation (i.e., forward or reverse). Protein annotations are shown in arrows. *bla_CTX-M-55_* is colored as indicated. The number of single-nucleotide polymorphisms (SNPs), based on the alignment of 2,888 core genes, between the isolates is shown. (d) The 446 plasmids were compared to each other in all non-redundant pairwise combinations via BLASTn. The resulting coverage and identity scores are binned and plotted. Bins are colored by frequency as indicated. Download FIG S4, TIF file, 1.7 MB. Copyright © 2022 Mahmud et al. 2022 Mahmud et al. https://creativecommons.org/licenses/by/4.0/ This content is distributed under the terms of the Creative Commons Attribution 4.0 International license.

### ESBLs are disseminated through distinct plasmid groups.

**FIG 5:**
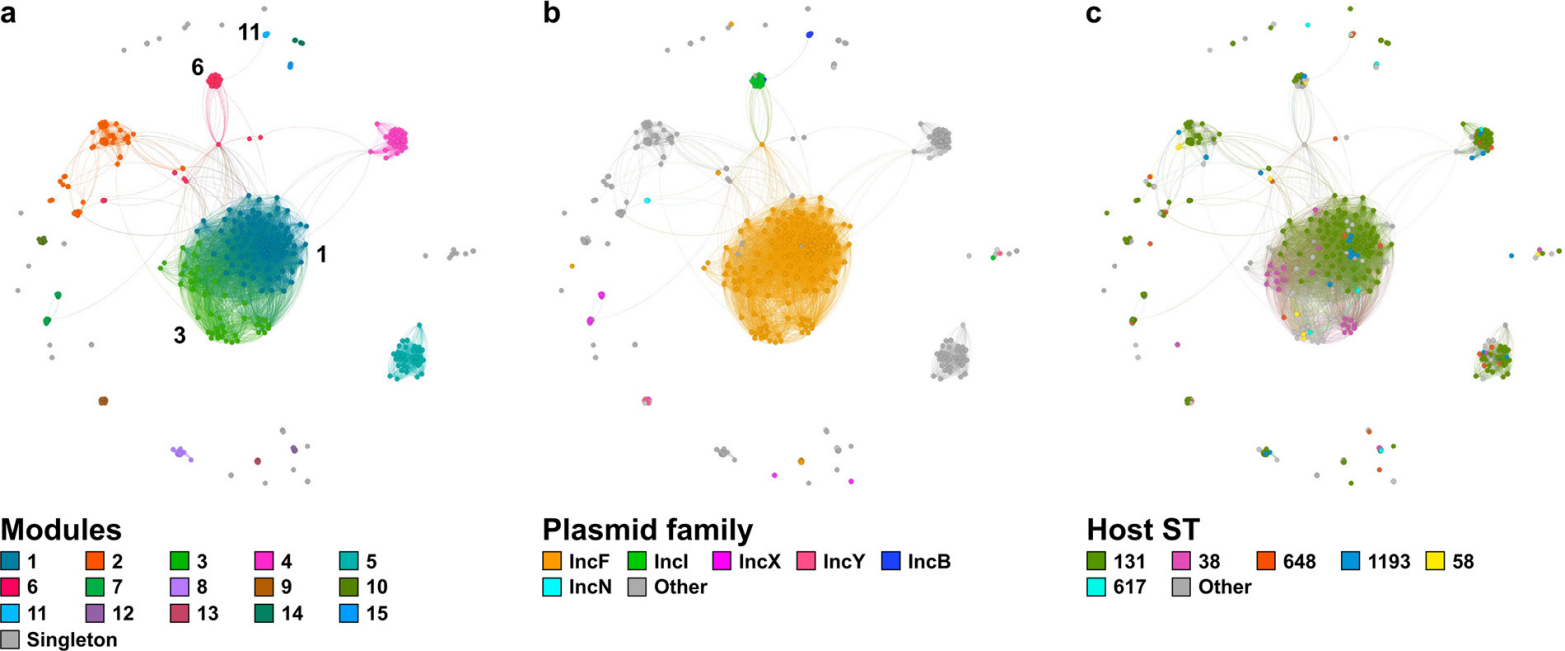
Clustering of plasmids by average-nucleotide identity (ANI). Similarity network of the 446 plasmids assembled from hybrid sequencing of the 149 ESBL E. coli isolates. Plasmids are represented by nodes. Pairs of nodes are connected with edges with weights corresponding to the ANI scores. The network is colored by identified modules (a), major replicon types (b), and major sequence types (STs) of the corresponding hosts (c).

The plastic genetic architecture of plasmids impedes efficient tracking of plasmid families responsible for dissemination of resistance genes based on high BLAST coverage and identity matches alone (39) (Fig. S4d). To address that, we implemented an average-nucleotide identity (ANI)-based clustering method that reveals groups of functionally related plasmids (40). Briefly, ANI scores were calculated for all pairwise combinations of the 446 plasmids. ANI values were filtered by coverage and transformed into edge weights to be used in a network with each plasmid represented by a node. After network spatialization, the clusters were identified via modularity optimization (see Materials and Methods). We identified 15 groups (hereafter referred to as modules) of related plasmids (Fig. 5a). The identified modules separate well by replicon types (Fig. 5b) and mobilization families (Fig. S5b), and primarily consisted of similarly-sized plasmids (Fig. S5a).

10.1128/msystems.00519-22.7 FIG S5 Average-nucleotide identity network of the plasmidic contigs. The network is colored by modules (a), MOB families (b), encoded extended-spectrum beta-lactamase (ESBL) (c), and presence of antibiotic resistance genes (d). Plasmids are represented by nodes. Pairs of nodes are connected with edges with weights corresponding to the ANI scores. In panel (a), the sizes of the nodes are proportional to the sizes of corresponding plasmids. Download FIG S5, TIF file, 2.6 MB. Copyright © 2022 Mahmud et al. 2022 Mahmud et al. https://creativecommons.org/licenses/by/4.0/ This content is distributed under the terms of the Creative Commons Attribution 4.0 International license.

Specific plasmid modules are significantly associated with different ESBL genes, presenting such modules as primary agents of horizontal spread of ESBLs. Module 6 was identified to be significantly enriched in *bla_CTX-M-15_* (*P* = 0.009, Chi-Square goodness of fit) (Fig. S5c). Module 6 comprises 36 plasmids belonging predominantly to the IncI group (Fig. 5b) and encoding the MOBP family of relaxases (Fig. S5b). Plasmids within module 6 were found in isolates representing 13 distinct sequence types (Fig. 5c), with only 36% (13/26) associated with ST131, suggesting a broader host range of this plasmid group relative to other modules. As before, *bla_CTX-M-15_* genes within this module were predominantly (71.4%, 5/7) encoded downstream of *ISEcp1*. Module 11 plasmids, on the other hand, were found to be significantly enriched in *bla_CTX-M-14_* (*P* = 4.8 × 10-4, Chi-Square goodness of fit); this module consists of 8 IncB plasmids with MOBP family of relaxases, primarily hosted by ST131 (4/8, 50%) and ST648 (3/8, 37.5%) E. coli isolates.

IncF family of plasmids have been hypothesized to be the primary carriers of ESBLs, particularly of *bla_CTX-M-15_* (8–11). Both antibiotic resistance genes (*P* = 0.0104, Chi-Square goodness of fit) and ESBLs (*P* = 2.35 × 10^−19^, Chi-Square goodness of fit) were enriched in IncF plasmids relative to other plasmid types (Fig. S5c and d). Interestingly, while all IncF plasmids tend to cluster together in our network (Fig. 5b), they were separated into two distinct modules (1 & 3) (Fig. 5a). Beyond sharing the replicon type, the 2 plasmid modules also commonly encode relaxases belonging to the MOBF family (Fig. S5b). However, the modules differ in numbers of member plasmids, and module 1 is the larger of the two and the largest module in the network, consisting of 94 plasmids; in comparison, module 3 has 44 plasmids. The sizes of the member plasmids differed significantly (*P* = 0.01, Mann-Whitney U Test) between modules 1 (mean = 117.4 Kb; median = 119.1 Kb) and 3 (mean = 100.2 Kb; median = 75.1 Kb). The 2 modules also have differing host ranges: while most of the module 1 plasmids are carried by ST131 E. coli (70/94, 74.5%), the 2 most common module 3 hosts were ST38 (16/44, 36.4%) and ST744 (5/44, 11.4%) (Fig. 5c). The 2 modules also vary in their associations with specific ESBL genotypes. While module 1 plasmids are highly enriched in *bla_CTX-M-15_* (*P* = 2.88 × 10-6, Chi-Square goodness of fit) and *bla_CTX-M-27_* (*P* = 4.64 × 10-13, Chi-Square goodness of fit) genes, module 3 was enriched in *bla_CTX-M-55_* (*P* = 3.16 × 10^−13^, Chi-Square goodness of fit) and *bla_CTX-M-14_* (*P* = 2.47 × 10^−10^, Chi-Square goodness of fit) (Fig. S5c). Notably, while *bla_CTX-M-15_* overall and within module 2 was predominantly associated with *ISEscp1*, the gene was primarily (64.3%, 9/14) associated with *IS26* within module 1. Analysis of the module 1 pangenome revealed the genetic variability within this group, devoid of a core (present in >99% of plasmids) set among the 1,092 total encoded genes (Fig. S6a). However, the majority of plasmids within this module (92.6%, 87/94) were found to encode the *ccdB*/*ccdA* toxin/antitoxin pair. Moreover, yet another toxin/antitoxin pair, *pemK*/*pemI*, was commonly (85.1%, 80/94) encoded within this module. Similarly, the genetic content of the module 3 plasmids was highly variable, lacking core genes among the 1,109 total coding sequences (Fig. S6b). Plasmid addiction systems were also identified in this module, with the *vapC*/*vapB* and *pemK*/*pemI* toxin/antitoxin pairs encoded in 21 (47.7%) and 13 (29.5%) of the member plasmids. These finding were further corroborated through our functional annotation of the encoded genes and GO term enrichment analysis within plasmid modules. Both modules 1 and 3 were significantly enriched in genes involved in conjugation (*P* = 8.85 × 10^−197^ and *P* = 2.34 × 10^−158^, respectively) and plasmid maintenance (*P* = 1.63 × 10^−179^ and *P* = 2.89 × 10^−16^, respectively), relative to other plasmid modules in our network. Taken together, the mobilizable nature of these 2 modules in addition to the enrichment of plasmid maintenance mechanisms likely underlie the widespread and stable presence of such plasmids within the diverse clinical E. coli population.

10.1128/msystems.00519-22.8 FIG S6 Pangenome plots for IncF plasmid modules. Variation in pan and core genome sizes are shown for modules 1 (a) and 3 (b). Download FIG S6, TIF file, 0.1 MB. Copyright © 2022 Mahmud et al. 2022 Mahmud et al. https://creativecommons.org/licenses/by/4.0/ This content is distributed under the terms of the Creative Commons Attribution 4.0 International license.

## DISCUSSION

The spread of ESBLs among clinical E. coli isolates has been rapid, and the rate of ESBL carriage among hospital-associated infections has been on the rise (4, 6, 7). This spread is primarily facilitated by the association of CTX-M family of ESBLs with MGEs, particularly transposable elements and plasmids (5). Here, we conducted an in-depth characterization of clinical ESBL E. coli isolates collected from a single tertiary care hospital, with a particular focus on the epidemiology of ESBLs and the MGEs underlying the spread of the resistance genes. We identified 8 distinct ESBLs within our set of 149 isolates, with large predominance of *bla_CTX-M-15_*, *bla_CTX-M-27_*, *bla_CTX-M-14_*, and *bla_CTX-M-55_* (Fig. 1b). This is mostly in agreement with prior reports (5); however, *bla_CTX-M-55_* has been primarily associated with animal reservoirs in Asia (41), and to our knowledge, its prevalence among human clinical E. coli isolates in North America has not been reported before. Interestingly, the 4 most common ESBL genes vary vastly in their genetic origins: while *bla_CTX-M-15_* is predominantly encoded in bacterial chromosomes, plasmids are the most common carriers of *bla_CTX-M-27_* and *bla_CTX-M-55_*, and *bla_CTX-M-14_* is split near-evenly between the two DNA types (Table S2). The reasons behind such contrasting distributions in genetic origins are unknown. Globally, *bla_CTX-M-15_* is the most predominant ESBL variant in most regions, with certain exceptions where the predominance of *bla_CTX-M-14_* is observed instead (5). *bla_CTX-M-27_* and *bla_CTX-M-55_* are not as common; however, their prevalence has been on the rise recently (5). Transfer of genes between plasmids and chromosomes via transposable elements is well established, and it has been suggested that over long periods of time plasmidic genes that are beneficial to the bacterial host are transferred to the chromosome (42–44). Thus, it may be speculated that varying prevalence of the ESBLs and the long-term chromosomal capture of plasmidic genes may explain the observed trends in their genetic origins; however, further investigation is necessary to test this hypothesis.

The commonplace transfer of ESBLs between plasmids and chromosomes is further demonstrated by identification of the resistance genes in conserved genetic contexts in both chromosomal and plasmidic contigs; presence of transposases within these conserved windows further supports their mobilizable nature. *ISEcp1* was the most common ESBL-associated transposase in our set (Fig. 3a to c) and more broadly (45). Beyond mediating mobilization, *ISEcp1* also serves as a promoter for the expression of the downstream ESBLs (46–49). Another prevalent ESBL-associated transposase in our isolate collection was *IS26*, which was found upstream of both *bla_CTX-M-15_* and *bla_CTX-M-27_* (Fig. 3d-f). The identified *IS26*-*bla_CTX-M-15_*-Δ*Tn2* has been reported before, and this genetic configuration was suggested to drive the amplification of *bla_CTX-M-15_* as part of the mechanism engendering non-carbapenemase carbapenem-resistance in *Enterobacterales* (35). We observed a similar instance of duplication of the *IS26*-*bla_CTX-M-15_*-Δ*Tn2* cassette in one of the plasmids (Fig. 3d). We also observed putative *IS26*-mediated duplication of *bla_CTX-M-27_* (Fig. 3e) and *ISEcp1*-mediated amplifications of *bla_CTX-M-14_* (Fig. S2b) and *bla_CTX-M-55_* (Fig. 3b); however, the clinical importance of these gene amplification events could not be established.

Plasmids are the primary elements of horizontal spread of ESBLs. Indeed, we identified a large network of plasmid sharing between phylogenetically distant isolates (Fig. 4b), including instances of within- and between-patient ESBL plasmid sharing (Fig. S4a and c). The highly variable genetic architecture of plasmids has been noted before (50), and extensive variations in encoded genes have been reported even for groups of environmentally-related plasmids (39). Similarly, our plasmid set also shows extensive sequence diversity (Fig. S4d). Such variability impedes the efficient tracking of plasmid groups within bacterial populations and identification of associated genetic patterns. To circumvent these challenges, we implemented a recently reported ANI-based network analysis of plasmids (40), which allowed us to identify clusters of circulating plasmids within the given E. coli population and determine the plasmid modules primarily responsible dissemination of ESBLs within this catchment area. We identified the enrichments of *bla_CTX-M-15_* in module 6, consisting of IncI plasmids occupying a wide host range, and module 1, comprising IncF plasmids mainly restricted to ST131 E. coli. However, while *bla_CTX-M-15_* was primarily found downstream of the *ISEcp1* transposase within module 6, the gene was mostly found in *IS26*-encoding genetic cassettes in module 1, suggesting that the 2 plasmid groups constitute 2 distinct *bla_CTX-M-15_* dissemination routes within the clinical E. coli population. IncI plasmids are described predominantly in European poultry E. coli isolates and have been primarily associated with the *bla_CTX-M-1_* spread (51–55). However, IncI plasmids carrying *bla_CTX-M-15_* have been previously identified in both human and animal E. coli isolates (36, 56). Moreover, recombination events involving *bla_CTX-M-15_* carried by IncI plasmids have been suggested to underlie the emergence of novel, hybrid CTX-M beta-lactamases (57). Similarly, module 11, consisting of IncB family of plasmids, was found to be enriched in *bla_CTX-M-14_* genes. IncB family, also labeled as IncO, are less prevalent among the ESBL E. coli population but have been reported to carry ESBLs (55, 58, 59); however, to our knowledge, this is the first reported instance of IncB/O plasmid-encoded *bla_CTX-M-14_*. Importantly, although we identified significant enrichment of *bla_CTX-M-14_* in IncB/O plasmids, the corresponding module 11 comprises only 8 plasmids, and as such the generalizability of this finding necessitates further investigations.

Previous reports establish IncF plasmids as the primary disseminators of ESBLs, most notably *bla_CTX-M-15_* and *bla_CTX-M-14_* (5, 15, 52, 59–65). Similarly, in our cohort, IncF plasmids were found to be significantly enriched in ESBLs relative to other plasmid families (Fig. S5c). Furthermore, our network analysis of plasmids indicated that the IncF plasmids exists within at least 2 major lineages (modules 1 & 3) with varying ESBL profiles and host preferences (Fig. 5b and Fig. S5c). While the association between ST131 E. coli and IncF plasmids has been noted (19, 60, 61, 66), only one of the IncF lineages in our cohort, module 1, was predominantly present in ST131 hosts. Module 3, despite being the smaller of the 2 IncF modules, had a broader host range, including 16 different STs (compared to 13 STs among the module 1 hosts), most commonly (36.4%) isolated from ST38 hosts. The 2 modules encompassed high gene content variability (Fig. S6); however, mobilization elements and plasmid addiction systems were commonly found within both plasmid modules, suggesting that there is a relatively constant backbone structure that includes elements essential for propagation and maintenance of these plasmids, while the accessory components of the plasmids undergo rapid and substantial diversification. Indeed, a recent genomic analysis of environmental and livestock IncF plasmids also identified a stable backbone structure within groups of related plasmids as well as a largely variable accessory sets of genes (39). Such diversity in the accessory gene content is suggested to be engendered by niche adaptations; however, there is a need for further investigations demonstrating the emergence of such variations and their roles in plasmid biology within variable environments.

Although we have performed comprehensive profiling of a large cohort of ESBL-producing strains, we acknowledge important study limitations. For one, our plasmid analysis only considered circular contigs within 1M bp. While such inclusion criteria would minimize the incidences of false positives (i.e., chromosomal sequences identified as plasmidic), it would omit any linear contigs of plasmidic origin. Thus, our plasmid analysis almost certainly didn’t capture the entire plasmidome of our sample set. However, given our hybrid sequencing and assembly approaches, most of the assembled contigs (72.0%, 601/835) could be accurately assigned to be of either chromosomal or plasmidic origins, and the majority of unassigned contigs were below 10,000 bp in length (median of 5,937 bp) (Fig. S7). Furthermore, the genetic nature (i.e., chromosome or plasmid) of 93.7% (164/175) of the ESBL-encoding contigs in our set could be determined. Given our aim to investigate the agents of ESBL spread, plasmid modules not associated with ESBL carriage were left underexplored. This was particularly true of the small plasmids, such as those found within modules 4 and 5, which were highly conserved and found within a diverse host range. The biological significance of these plasmids warrants further investigations, and our extensive sequencing and gene enrichment data (Data Set S1, Sheet B) will facilitate such future explorations. Furthermore, given that all bacterial isolates in the study originated from a single hospital system, future investigations focusing on more diverse geographical settings are necessary to determine the generalizability of our findings. Lastly, we also explicitly focused on clinical ESBL E. coli isolates given the growing importance of these organisms; as such, the broader implications of our findings regarding the nature and epidemiology of the described MGEs in non-ESBL clinical isolates or community-associated E. coli strains need additional validation.

10.1128/msystems.00519-22.9 FIG S7 Sizes of the unassigned contigs. Histogram depicting the size distribution of the linear contigs shorter than 1 Mb. Bin size is 10 Kb. Download FIG S7, TIF file, 0.3 MB. Copyright © 2022 Mahmud et al. 2022 Mahmud et al. https://creativecommons.org/licenses/by/4.0/ This content is distributed under the terms of the Creative Commons Attribution 4.0 International license.

10.1128/msystems.00519-22.10 Data Set S1 (Sheet A) Identified ESBLs in all 149 isolates. (Sheet B). Analysis of GO term enrichment in plasmid modules. Download Data Set S1, XLSX file, 0.8 MB. Copyright © 2022 Mahmud et al. 2022 Mahmud et al. https://creativecommons.org/licenses/by/4.0/ This content is distributed under the terms of the Creative Commons Attribution 4.0 International license.

In conclusion, we conducted an in-depth genomic analysis of infectious ESBL E. coli isolates from a single tertiary care hospital. Our analysis revealed the major ESBLs in circulation within the given population and identified MGEs associated with major resistance genes. Our high-quality genomic assemblies enabled us to characterize a nearly complete set of plasmids and detect clusters of related plasmids that are primarily responsible for the spread of ESBLs within the clinical E. coli population. Given the global ubiquity of ESBL E. coli colonization (1), similar network analyses of non-clinical isolates are warranted to investigate the spread of the identified plasmid groups outside of nosocomial settings.

## MATERIALS AND METHODS

### Sample collection.

All specimens and data used in this study were retrospective and collected during routine clinical care; as a result, this study was approved by the Washington University in St. Louis Human Research Protection Office with a waiver of informed consent.

The study was conducted at a 1250-bed, tertiary care hospital in St. Louis, Missouri. The hospital’s Medical Informatics database was queried to retrospectively identify all blood and urine cultures positive for “ESBL-like” E. coli from 2016–2019. Patients with urine and/or blood cultures positive for ESBL-like E. coli were included in the study cohort if they met the following criteria:

1. Admitted to a medicine, oncology, or medical ICU ward during the study period.

2. ESBL E. coli isolate was collected from a clinical specimen within 24 h prior to admission to the study ward through 24 h after discharge from the study ward.

For patients with >1 blood or urine specimens positive for E. coli during the study period, specimens of the same type (i.e., urine or blood) collected within 14 days of a prior positive specimen were excluded.

ESBL-like was defined as phenotypic susceptibility suggestive of an ESBL-producing strain: resistant to first-generation cephalosporins, resistant to at least one third-generation cephalosporin (with ceftazidime and ceftriaxone being routinely tested), and susceptible to cephamycins (with cefotetan being routinely tested). Antibiotic susceptibility testing (AST) was performed on all isolates selected for inclusion in the study cohort.

AST was performed on all isolates according to the Clinical and Laboratory Standards Institute M100 document. In short, an 18-h culture of each organism on tryptic soy agar with 5% sheep’s blood (Hardy Diagnostics) was used to make a 0.5 McFarland standard in saline. This suspension was inoculated on Mueller-Hinton agar (Hardy Diagnostics) in such a way to create a lawn of growth. Antibiotic disks (Hardy Diagnostics and BD, Franklin) were dropped on the Mueller-Hinton agar and incubated for 16–18 h. Each organism was tested against the following antibiotics: ampicillin (10 μg), cefazolin (30 μg), cefotetan (30 μg), ceftazidime (30 μg), ceftriaxone (30 μg), cefepime (30 μg), meropenem (10 μg), ertapenem (10 μg), imipenem (10 μg), cetolozane-tazobactam (30 μg/10 μg), ceftazidime-avibactam (30 μg/20 μg), ampicillin-sulbactam (20 μg), ciprofloxacin (5 μg), doxycycline (30 μg), gentamicin (10 μg), amikacin (30 μg), nitrofurantoin (300 μg), cefiderocol (30 μg), piperacillin-tazobactam (110 μg), fosfomycin (200 μg), colistin (10 μg), aztreonam (30 μg), trimethoprim-sulfamethoxazole (23.75 μg/1.25 μg), and tigecycline (15 μg). Zone sizes were measured using a metric ruler, and interpretations were given based on the M100 guidelines (67).

### Whole-genome sequencing and *de novo* genome assembly.

Where appropriate, the parameters used for the computational tools are provided parenthetically. Isolates were grown overnight on tryptic soy agar with 5% sheep’s blood (Hardy Diagnostics), and colonies were collected and resuspended in deionized water. Total genomic DNA from each suspension was isolated using the QIAamp BiOstic Bacteremia DNA Kit (Qiagen) and quantified using the Qubit dsDNA HS and BR Assay Kits (Invitrogen). For each isolate, 0.5 ng of the extracted DNA was used as the input for Illumina library preparation using the Nextera kit (Illumina) (68). Libraries were pooled and sequenced on the NovaSeq 6000 platform (Illumina) to obtain 2 × 150 bp reads. The reads were demultiplexed by index pair, quality trimmed by Trimmomatic (v0.38, SLIDINGWINDOW:4:20, LEADING:10, TRAILING:10, MINLEN:60) (69), and decontaminated using Deconseq (v4.3, -dbs hsref38) (70).

All isolates were also sequenced using the Oxford Nanopore Technologies long-read sequencing platform. Total genomic DNA was extracted as before, but the lysates in PowerBead Tubes were vortexed only for 90 s to yield high molecular weight DNA fragments. For each isolate, 1 μg of DNA was used to prepare sequencing libraries using the NEBNext kit (New England BioLabs) and sequenced using the MinION platform. The output fastq files were filtered using Filtlong (v2.0, –min_length 1000, –keep_percent 95, –target_bases 500000000) (https://github.com/rrwick/Filtlong) and used for *de novo* genome assembly using Flye (v2.8.1, –plasmids) (71). The resulting assemblies were further corrected with the processed Illumina reads using Pilon (72). The resulting contigs were determined to be chromosomal if their length exceeded 1M bp, and circular contigs <1M bp in length were assigned to be plasmidic.

### Phylogenetic analysis.

Assembled contigs were annotated using Prokka (v1.14.5, -mincontiglen 200, -force, -rnammer) (73). The resulting gff files were used to identify the core genome set and construct a corresponding alignment using Roary (v.3.13.0, -g 500000, -e) (74). The core genome alignment was used to construct a maximum likelihood tree with FastTree (v2.1.10, -nt, -gtr) (75). The resulting newick file was visualized in iTOL (76). *In silico* MLST was performed via the MLST tool (v2.19, –scheme ecoli_4) (https://github.com/tseemann/mlst) using the Achtman scheme. The pairwise SNP counts were generated by using the core genome alignment as the input for snp-dists (v0.8.2) (https://github.com/tseemann/snp-dists). Within groups of highly related isolates (as determined through the core genome SNP counts), the processed Illumina reads for each isolate were mapped to a common reference (i.e., assembled complete chromosomal contig of one of the isolates) using snippy (v4.4.3) (https://github.com/tseemann/snippy); the resulting alignment was similarly used as the input for snp-dists (v0.8.2) to determine the pairwise SNP counts.

### ESBL identification.

Encoded ESBLs were identified using AMRFinder (v3.10.16) on the Prokka-generated gff files. Only for the determination of the genetic origins (chromosomal or plasmidic) of the identified ESBLs, the linear contigs <1M bp in size were assigned as putative plasmids if the corresponding isolate chromosomal contig was complete (i.e., circular); however, such putatively plasmidic contigs were not considered for the plasmid sharing and ANI-based network analyses. Transposases neighboring ESBLs were first annotated through Prokka and subsequently validated by aligning the protein sequences against the ISfinder database (77). The analysis and visualization of the genomic contexts of ESBL genotypes was done using Easyfig (v2.2.2) (78).

### Plasmid typing and functional annotation.

For our plasmid analysis, we only included circular contigs <1M bp in size, resulting in 446 plasmidic contigs. Replicon typing of the plasmid sequences was done using PlasmidFinder (v2.0.1) (79) using the *Enterobacterales* database with 95% minimum identity and 60% minimum coverage. The encoded relaxases were identified and classified through MOBscan (80) using the Prokka-generated faa files. The functional annotation of the plasmid-encoded genes was done using Blast2GO (81) using the default parameters. For the analysis of plasmid sharing, pairs of plasmids were aligned to each other in all possible, non-redundant combinations using the default BLASTn parameters.

### Plasmid cluster analysis.

The plasmid clustering method was first reported elsewhere (40) and was used here with modifications. The pairwise ANI values were calculated for all nonredundant plasmid combinations using fastANI (v1.33) (82) with the following parameters: fragLen 250 and minFraction 0.5. A custom Python script was used to remove self-comparisons and average the values for reciprocal pairs. The resulting pairwise ANI values were transformed to edge weights to be used in the subsequent plasmid network as follows:

(1) $$\text{Edge weight}=\frac{1}{1\;+\;100(1\;-\text{ ANI}/100)}$$

The plasmid network was constructed using the linear logarithmic version of ForceAtlas2 (83) within Gephi. In this network, plasmids were represented as nodes, and pairs of nodes with non-zero ANI scores were linked together with edges weights calculated as above (Equation 1). ForceAtlas2 was used with the following parameters: approximation 1.2, scaling 0.1, gravity 1.0, and edge weight influence 1.0. The plasmid clusters within the spatialized network were identified by using the modularity optimization algorithm built within Gephi (84, 85). Lastly, modules with fewer than 4 plasmids were excluded.

### Statistical analysis.

The enrichment of genes and GO terms in plasmid modules and sequence types was done using the Chi-Square goodness of fit test. In each instance, the expected values were calculated to be proportional to the number of plasmids withing the tested module relative to the total number of plasmids. The observed and expected values were used as inputs for the “chisquare” function within the SciPy library in Python. The resulting *P*-values were adjusted for multiple comparisons using the Bonferroni method in the “p.adjust” function within the stats library in R. The enrichment of ESBLs in STs, and ESBLs and ARGs in IncF plasmids were also tested as described.

### Data availability.

All isolate assemblies as well as the sequencing data from Illumina and Oxford Nanopore Technologies platforms are available in NCBI GenBank under BioProject accession no. PRJNA824420, which includes short- (accession no. SRR18678975-9123) and long-read (accession no. SRR18681602-750) SRA, as well as the BioSample hybrid assemblies (accession no. SAMN27404312-460) for all 149 isolates in the study.
