# Supplementary material for: Epidemiology of Plasmid Lineages Mediating the Spread of Extended-Spectrum Beta-Lactamases among Clinical Escherichia coli
Source: mSystems. 2022 Aug 22;7(5):e00519-22. doi: 10.1128/msystems.00519-22 (PMC9601178; doi:10.1128/msystems.00519-22)
Supplement: TABLE S1 [file msystems.00519-22-s0008.docx]

| **Total isolates** | 149 |
| --- | --- |
| **Isolate origin (%)** |  |
| Urine (69.8%) | 104 |
| Blood (30.2%) | 45 |
| **Collection ward (%)** |  |
| Medical (84.6%) | 126 |
| Oncology (15.4%) | 23 |
|  |  |
| **Total patients** | 129 |
| **Isolates/patient** |  |
| 1 | 111 |
| 2 | 16 |
| 3 | 2 |

**Table S1. Isolates and patients involved in the study.**
