# Supplementary material for: Epidemiology of Plasmid Lineages Mediating the Spread of Extended-Spectrum Beta-Lactamases among Clinical Escherichia coli
Source: mSystems. 2022 Aug 22;7(5):e00519-22. doi: 10.1128/msystems.00519-22 (PMC9601178; doi:10.1128/msystems.00519-22)
Supplement: TABLE S2 [file msystems.00519-22-s0009.docx]

| **Gene (total)** | **Chr (%)** | **Plas (%)** | **Uniden (%)** |
| --- | --- | --- | --- |
| *bla_CTX-M-15_* (81) | 60 (74.1%) | 20 (24.7%) | 1 (1.2%) |
| *bla_CTX-M-14_* (39) | 20 (51.3%) | 19 (48.7%) | 0 |
| *bla_CTX-M-27_* (31) | 1 (3.2%) | 30 (96.8%) | 0 |
| *bla_CTX-M-55_* (18) | 1 (5.6%) | 17 (94.4%) | 0 |
| *bla_CTX-M_* (3) | 3 (100%) | 0 | 0 |
| *bla_TEM-29_* (1) | 0 | 1 (100%) | 0 |
| *bla_SHV-7_* (1) | 0 | 1 (100%) | 0 |
| *bla_CTX-M-24_* (1) | 0 | 0 | 1 (100%) |

**Table S2. Genetic origin (chromosomal vs. plasmidic) of the identified extended spectrum beta-lactamases.**
