# Supplementary figures and images for: Epidemiology of Plasmid Lineages Mediating the Spread of Extended-Spectrum Beta-Lactamases among Clinical Escherichia coli
Source: mSystems. 2022 Aug 22;7(5):e00519-22. doi: 10.1128/msystems.00519-22 (PMC9601178; doi:10.1128/msystems.00519-22)

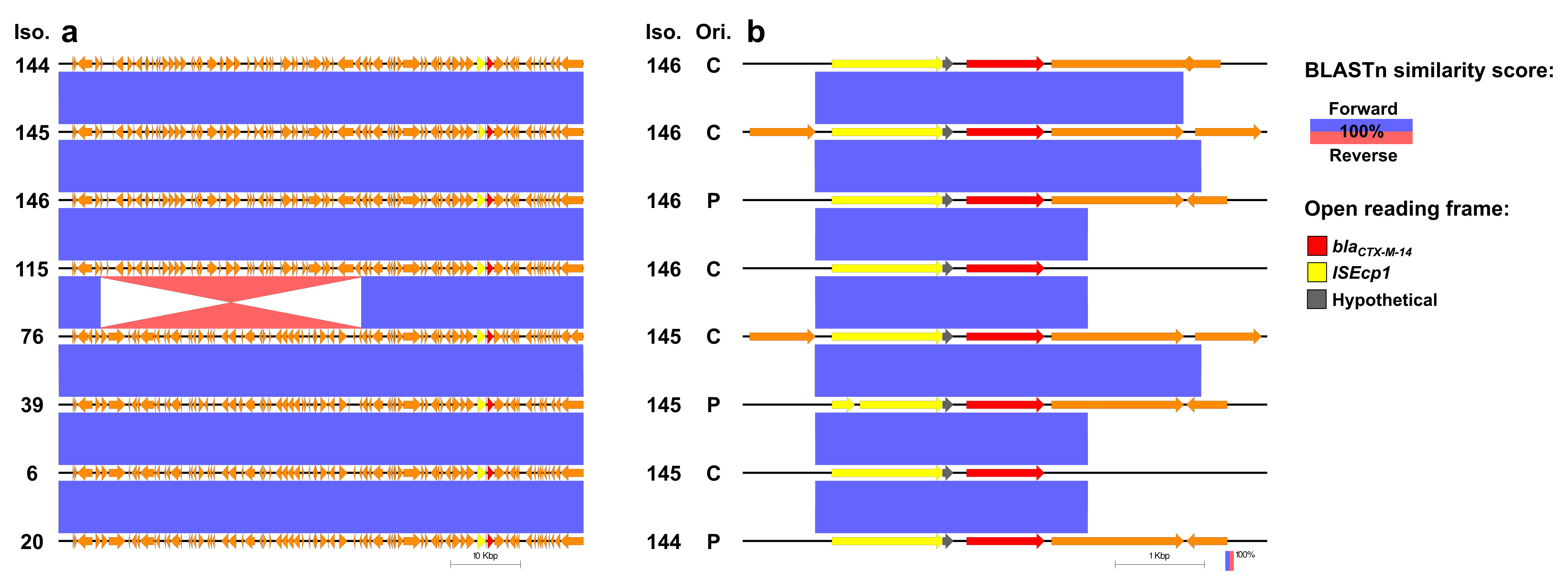

Supplement: FIG S2 [file msystems.00519-22-s0002.tif]

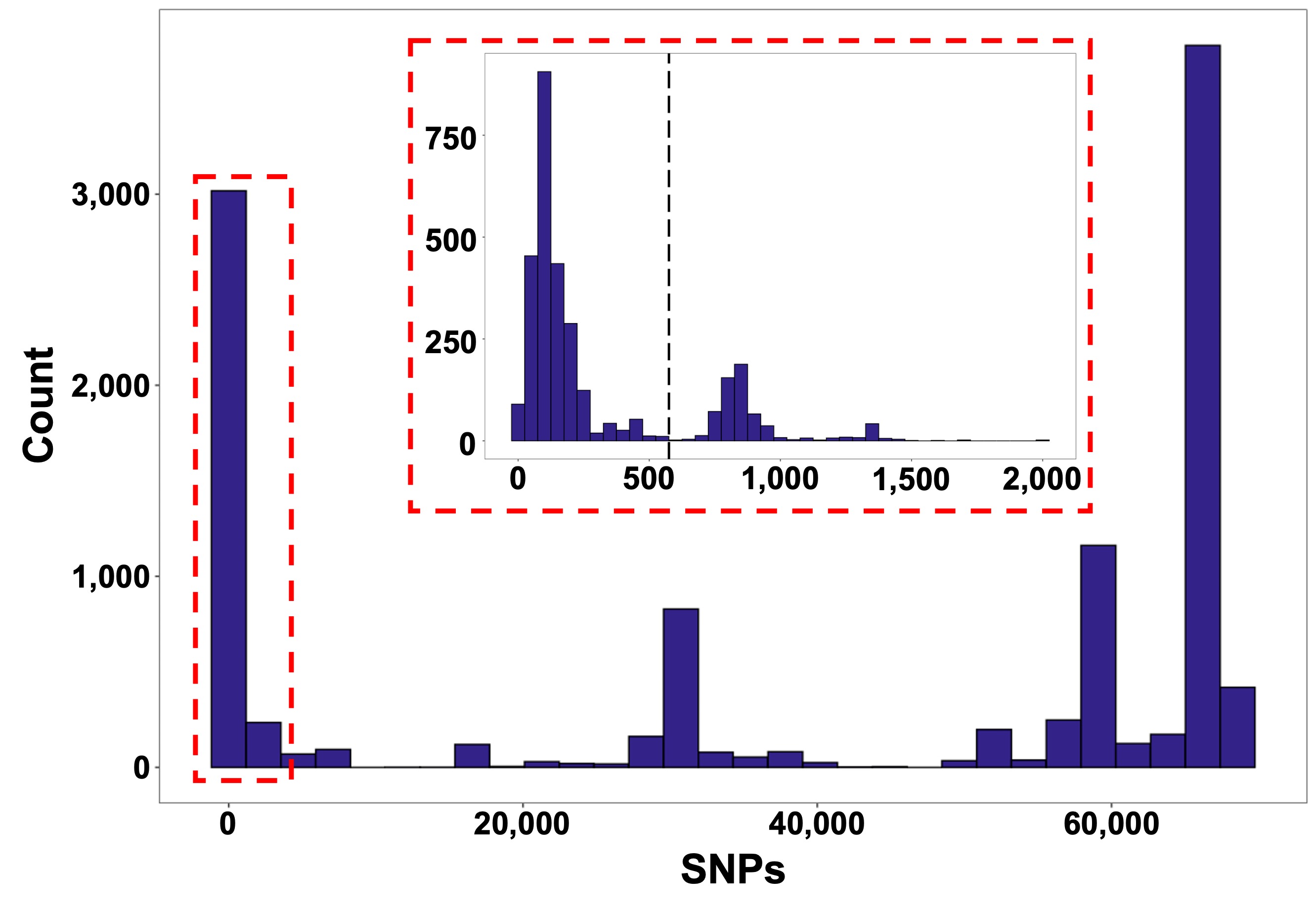

Supplement: FIG S3 [file msystems.00519-22-s0003.jpg]

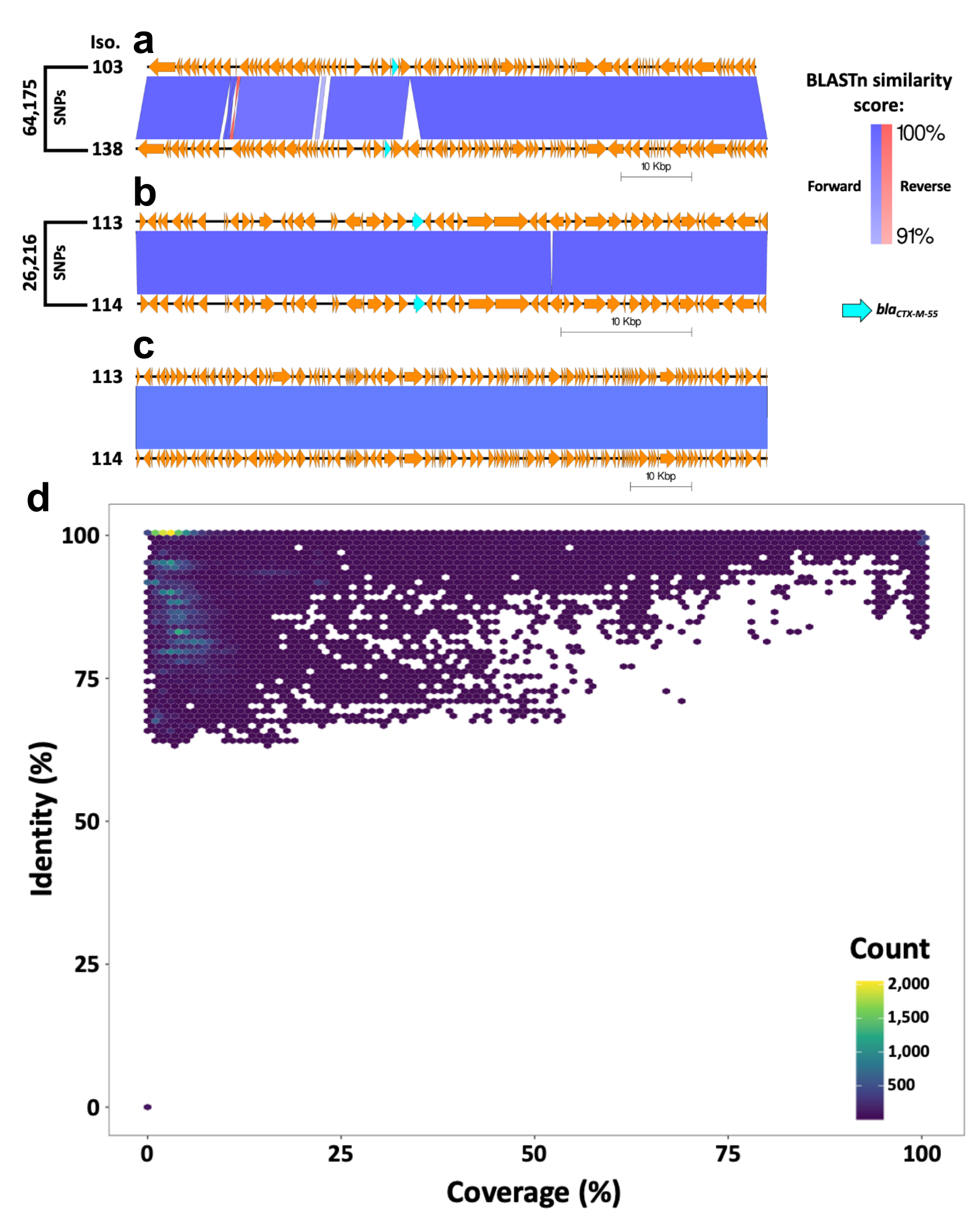

Supplement: FIG S4 [file msystems.00519-22-s0004.tif]

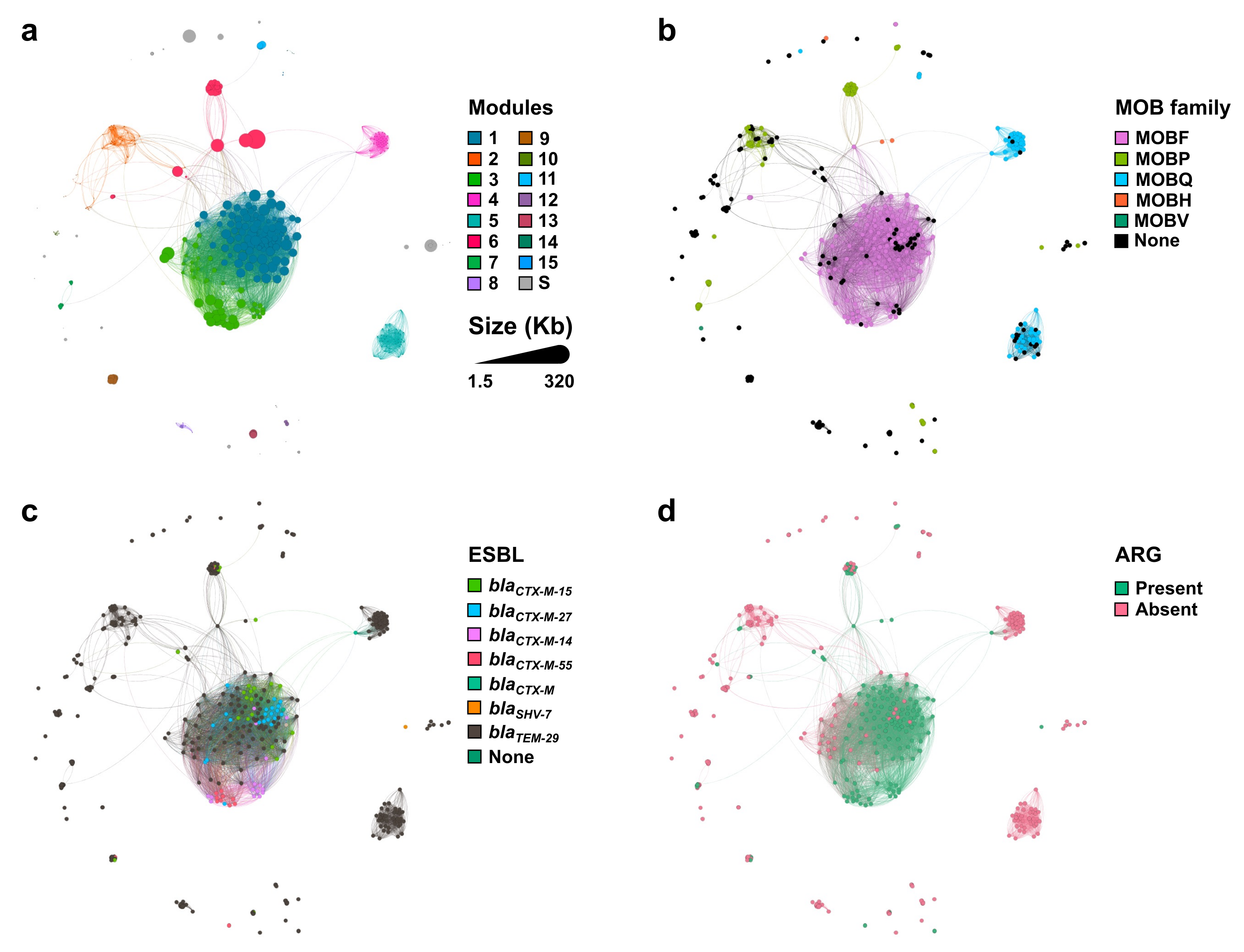

Supplement: FIG S5 [file msystems.00519-22-s0005.tif]

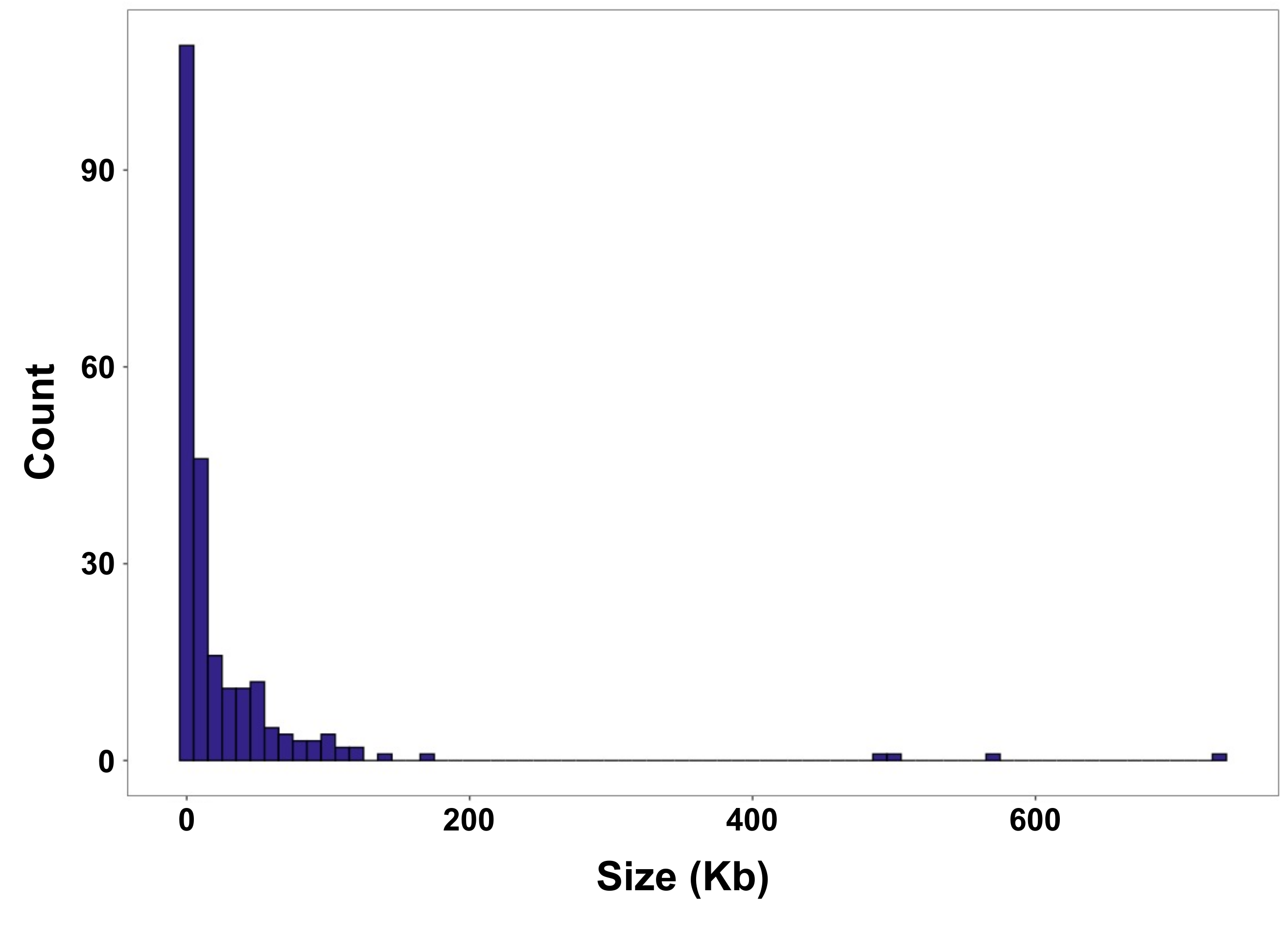

Supplement: FIG S7 [file msystems.00519-22-s0007.tif]
